# Dynamin-Related Protein 1 Inhibitors Protect against Ischemic Toxicity through Attenuating Mitochondrial Ca2+ Uptake from Endoplasmic Reticulum Store in PC12 Cells

**DOI:** 10.3390/ijms15023172

**Published:** 2014-02-21

**Authors:** Ye Tian, Bin Li, Wen-Zhen Shi, Ming-Ze Chang, Ge-Juan Zhang, Zheng-Li Di, Yong Liu

**Affiliations:** 1 Department of Neurology, Xi’an Central Hospital, Xi’an Jiaotong University School of Medicine, Xi’an 710003, Shaanxi, China; E-Mails: xijiaodatianye@163.com (Y.T.); xijiaodalibin@163.com (B.L.); xijiaodashiwz@163.com (W.-Z.S.); xijiaodachangmz@163.com (M.-Z.C.); xijiaodazhanggj@163.com (G.-J.Z.); xijiaodadizl@163.com (Z.-L.D.); 2 Institute of Neurobiology, Key Laboratory of Environment and Genes Related to Diseases of Education Ministry, Xi’an Jiaotong University School of Medicine, Xi’an 710061, Shaanxi, China

**Keywords:** stroke, Drp-1, calcium, mitochondria, endoplasmic reticulum

## Abstract

Intracellular calcium homeostasis disorder and mitochondrial dysfunction are involved in many acute and chronic brain diseases, including ischemic brain injury. An imbalance in mitochondrial fission and fusion is one of the most important structural abnormalities found in a large number of mitochondrial dysfunction related diseases. Here, we investigated the effects of mitochondrial division inhibitor A (mdivi A) and mdivi B, two small molecule inhibitors of mitochondrial fission protein dunamin-related protein 1 (Drp-1), in neuronal injury induced by oxygen-glucose deprivation (OGD) in PC12 cells. We found that mdivi A and mdivi B inhibited OGD-induced neuronal injury through attenuating apoptotic cell death. These two inhibitors also preserved mitochondrial function, as evidenced by reduced reactive oxygen species (ROS) generation and cytochrome c release, as well as prevented loss of mitochondrial membrane potential (MMP). Moreover, mdivi A and mdivi B significantly suppressed mitochondrial Ca^2+^ uptake, but had no effect on cytoplasmic Ca^2+^ after OGD injury. The results of calcium imaging and immunofluorescence staining showed that Drp-1 inhibitors attenuated endoplasmic reticulum (ER) Ca^2+^ release and prevented ER morphological changes induced by OGD. These results demonstrate that Drp-1 inhibitors protect against ischemic neuronal injury through inhibiting mitochondrial Ca^2+^ uptake from the ER store and attenuating mitochondrial dysfunction.

## 1. Introduction

Stroke is the second most common cause of death and a significant cause of long-term disability worldwide. The number of global deaths caused by stroke is projected to rise to 6.5 million in 2015 and 7.8 million in 2030 [1]. The mechanisms of ischemic stroke are considered to be related to hyper-activation of ionotropic and metabotropic glutamate receptors, massive influx of calcium ions, over-production of reactive oxygen species (ROS) and over-activation of Ca^2+^-dependent lethal enzymes, but the exact molecular mechanisms are not fully understood [2,3].

Brain ischemic injury results in O_2_ and ATP depletion and toxic metabolites buildup, which is followed by re-establishment of blood flow through increased generation of reactive oxygen and nitrogen species [2,4]. Mitochondria play essential roles in energy metabolism, and increasing evidence suggests that structural and functional abnormalities in mitochondria are involved in the regulation of the cell death pathways in response to ischemic brain injury [5]. An imbalance in mitochondrial division and mitochondrial fusion is one of the most important structural abnormalities found in a large number of neurological diseases, and the *GTPase* gene dynamin-related protein 1 (Drp-1) is considered to be a key molecular in regulating mitochondrial fission [6]. Drp-1 activation leads to abnormalities in mitochondrial structure and function, inhibits ATP generation and activates pro-apoptotic signaling cascades [6]. A recent study showed that embryos of Drp-1 knockout mouse died on days 11 to 12 [7], and experiments using pharmacological inhibitors seem to be an ideal strategy. In the present study, small molecule inhibitors were used to investigate Drp-1 dependent mitochondrial death pathways in oxygen-glucose deprivation (OGD) in PC12 cells. We also examined the changes of intra-cellular calcium homeostasis to address the potential underlying mechanisms.

## 2. Results

### 2.1. Effects of Drp-1 Inhibitors on Mitochondrial Dynamic Proteins

**Figure 1.**
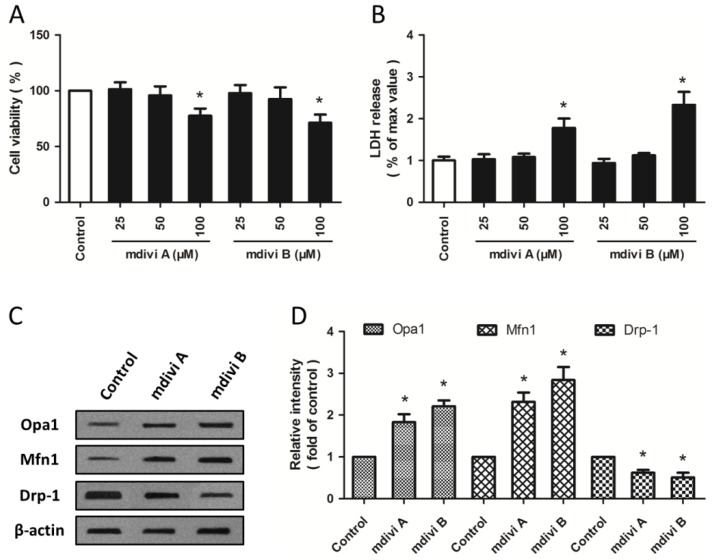
Effects of Drp-1 inhibitors on mitochondrial dynamic proteins. PC12 cells were treated with mdivi A or mdivi B at different concentrations (25, 50 or 100 μM) for 24 h. Cell viability was measured with the WST assay (**A**); and cytotoxicity was measured with the LDH assay (**B**); PC12 cells were treated with mdivi A (50 μM) or mdivi B (50 μM) for 24 h, and the expression of Opa1, Mfn1 and Drp-1 were detected by western blot (**C**) and calculated (**D**). The data were represented as means ± SD from five experiments. * *p* < 0.05 *vs.* control.

Cultured PC12 cells were treated with mdivi A or mdivi B in different concentrations (25, 50 and 100 μM) to examine the possible toxic effects of mdivi compounds at higher concentrations. As shown in Figure 1A, the cell viability was decreased by mdivi A (100 μM) and mdivi B (100 μM), whereas mdivi compounds at low concentrations (25 or 50 μM) had no effect on cell viability. These results were confirmed by lactate dehydrogenase (LDH) release assay (Figure 1B). Furthermore, western blot was used to detect the expression of mitochondrial dynamic proteins (Figure 1C). Both mdivi A and mdivi B significantly increased the expression of optic atrophy type 1 (Opa1) and mitofusin 1 (Mfn1), two fusion related mitochondrial dynamic proteins, and decreased the expression of Drp-1 (Figure 1D). All these data indicated that mdivi A and mdivi B at 50 μM differentially regulated mitochondrial dynamics-related proteins, but had no toxic effects in PC12 cells.

### 2.2. Drp-1 Inhibitors Reduce Ischemic Toxicity in PC12 Cells

**Figure 2.**
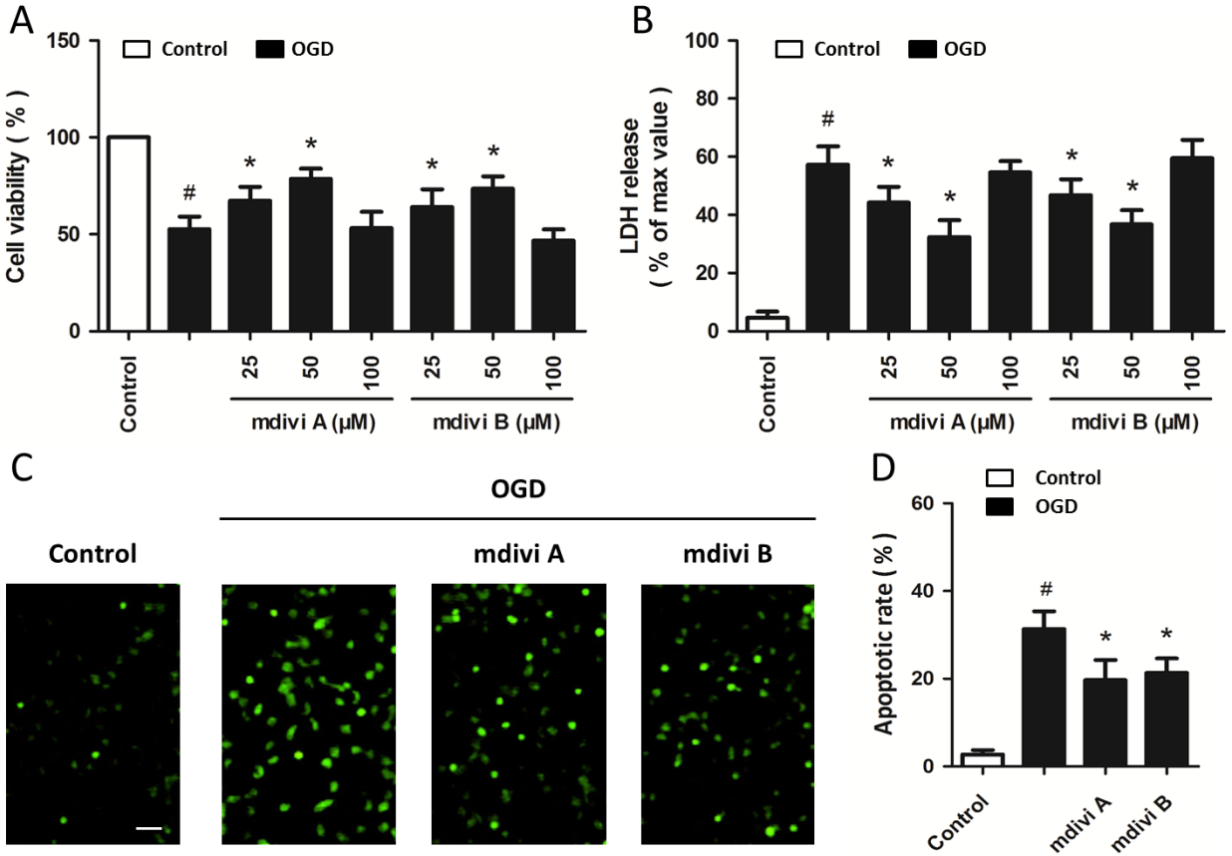
Drp-1 inhibitors reduce ischemic toxicity in PC12 cells. PC12 cells were pretreated with mdivi A or mdivi B at different concentrations (25, 50 or 100 μM) for 30 min before oxygen-glucose deprivation (OGD) injury. Cell viability was measured with the WST assay (**A**); and cytotoxicity was measured with the LDH assay (**B**); PC12 cells were pretreated with mdiviA (50 μM) or mdivi B (50 μM) for 30 min before OGD injury. Apoptotic cell death was detected by TUNEL staining (**C**) and calculated (**D**). Scale bar: 40 μm. The data were represented as means ± SD from five experiments. ^#^ *p* < 0.05 *vs.* control. * *p* < 0.05 *vs.* OGD.

Cultured PC12 cells were pretreated with mdivi A or mdivi B in different concentrations (25, 50 and 100 μM) for 30 min before OGD and cell viability was measured at 24 h after reoxygenation. It was found that the cell viability increased with the concentrations of mdivi A and mdivi B added, although 100 μM mdivi A or mdivi B was not effective compared with OGD injured cells (Figure 2A). LDH assay also showed that pretreatment with mdivi A and mdivi B (25 and 50 μM) induced a significant decrease in LDH release after OGD insult (Figure 2B). Moreover, terminal deoxynucleotidyl transferase dUTP nick end labeling (TUNEL) staining was used to determine the effects of mdivi A and mdivi B on OGD-induced apoptotic cell death (Figure 2C). As shown in Figure 2D, the OGD-induced increase of TUNEL-positive cells was significantly decreased by mdivi A and mdivi B pretreatment, indicating the anti-apoptotic activity of Drp-1 inhibition.

### 2.3. Drp-1 Inhibitors Attenuate Mitochondrial Dysfunction

**Figure 3.**
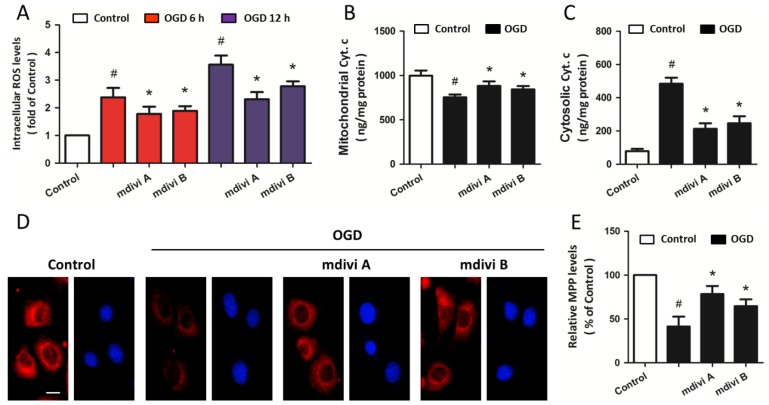
Drp-1 inhibitors attenuate mitochondrial dysfunction. PC12 cells were pretreated with mdivi A (50 μM) or mdivi B (50 μM) for 30 min before OGD injury. Intracellular ROS levels were measured at 6 and 12 h after OGD initiation (**A**); release of cytochrome c into the cytoplasm was determined by an immunoassay kit after subcellular fraction preparation (**B**,**C**); The loss of mitochondrial membrane potential (MMP) was determined by Rh123 staining (**D**) and calculated (**E**). Scale bar: 10 μm. The data were represented as means ± SD from five experiments. ^#^ *p* < 0.05 *vs.* control. * *p* < 0.05 *vs.* OGD.

In order to investigate the effects of Drp-1 inhibitors on mitochondrial dysfunction, PC12 cells were pretreated with 50 μM mdivi A or 50 μM mdivi B based on the results mentioned above. Exposure to OGD insults resulted in an increase in intracellular ROS generation at both 6 and 12 h after OGD initiation, and pretreatment with mdivi A or mdivi B significantly reduced the ROS overproduction (Figure 3A). As shown in Figure 3B,C, mdivi A and mdivi B pre-treatment also attenuated release of cytochrome c into the cytoplasm after OGD injury. In addition, the MMP was determined by using the fluorescent dye Rh123. OGD insults resulted in a decline in red fluorescence intensity in the cytoplasm, and Drp-1 inhibitors partially prevented the loss of MMP (Figure 3D,E). These data suggest that Drp-1 may be upstream of mitochondrial dysfunction after ischemic neuronal injury in our *in vitro* model.

### 2.4. Effects of Drp-1 Inhibitors on Mitochondrial Ca^2+^ Uptake

**Figure 4.**
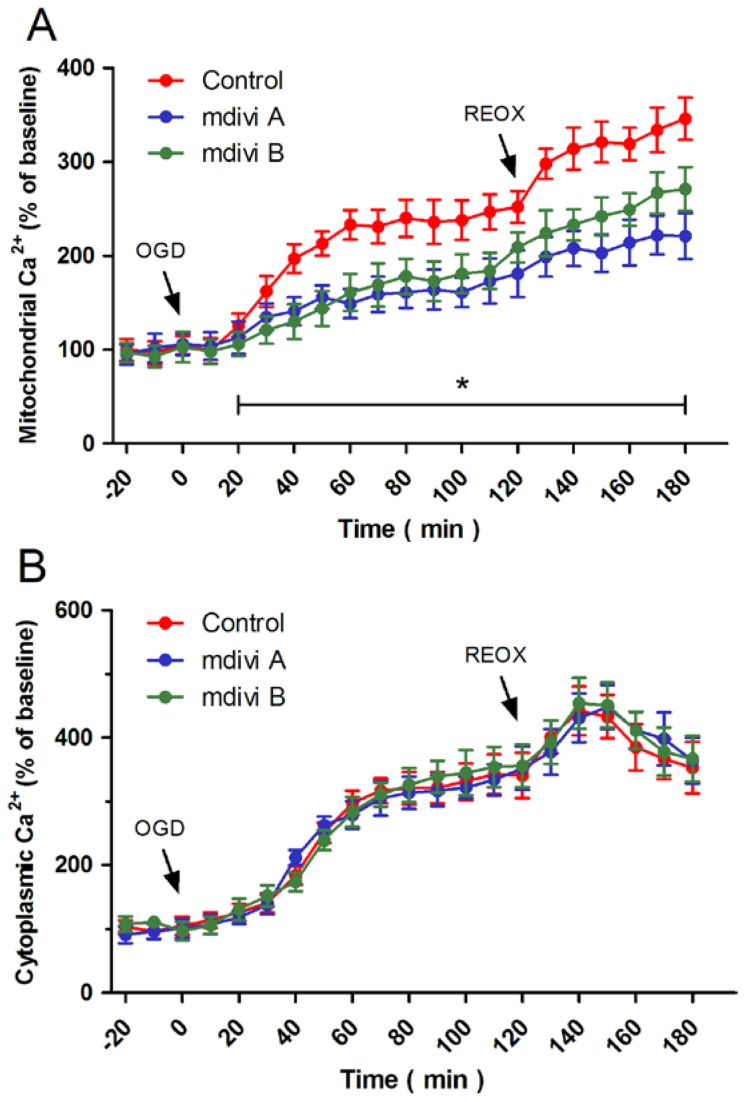
Effects of Drp-1inhibitors on mitochondrial Ca^2+^ uptake. PC12 cells were pretreated with mdivi A (50 μM) or mdivi B (50 μM) for 30 min before OGD injury. The mitochondrial Ca^2+^ concentration (**A**) and cytoplasmic Ca^2+^ concentration (**B**) were measured up to 180 min after OGD initiation. The data were represented as means ± SD from five experiments. * *p* < 0.05.

Calcium imaging was used to assess the relationship between Drp-1 inhibitors-induced protection and intracellular Ca^2+^ homeostasis. Figure 4A shows dynamic changes of mitochondrial Ca^2+^, expressed as a percentage of the baseline for up to 180 min following OGD initiation. OGD triggered a rapid rise in mitochondrial Ca^2+^, which was further increased by re-oxygenation, indicating an enhanced mitochondrial Ca^2+^ uptake by OGD and reoxygenation. Pretreatment with mdivi A or mdivi B obviously inhibited mitochondrial Ca^2+^ uptake at both ischemic and reoxygenation phases. Intriguingly, these two Drp-1 inhibitors had no effect on the increase of cytoplasmic Ca^2+^ induced by OGD and reoxygenation (Figure 4B). These results indicate that the Drp-1 inhibitors-induced attenuation of mitochondrial Ca^2+^ uptake might be mediated by a cytoplasmic Ca^2+^-independent mechanism.

### 2.5. Effects of Drp-1 Inhibitors on ER Ca^2+^ Release

**Figure 5.**
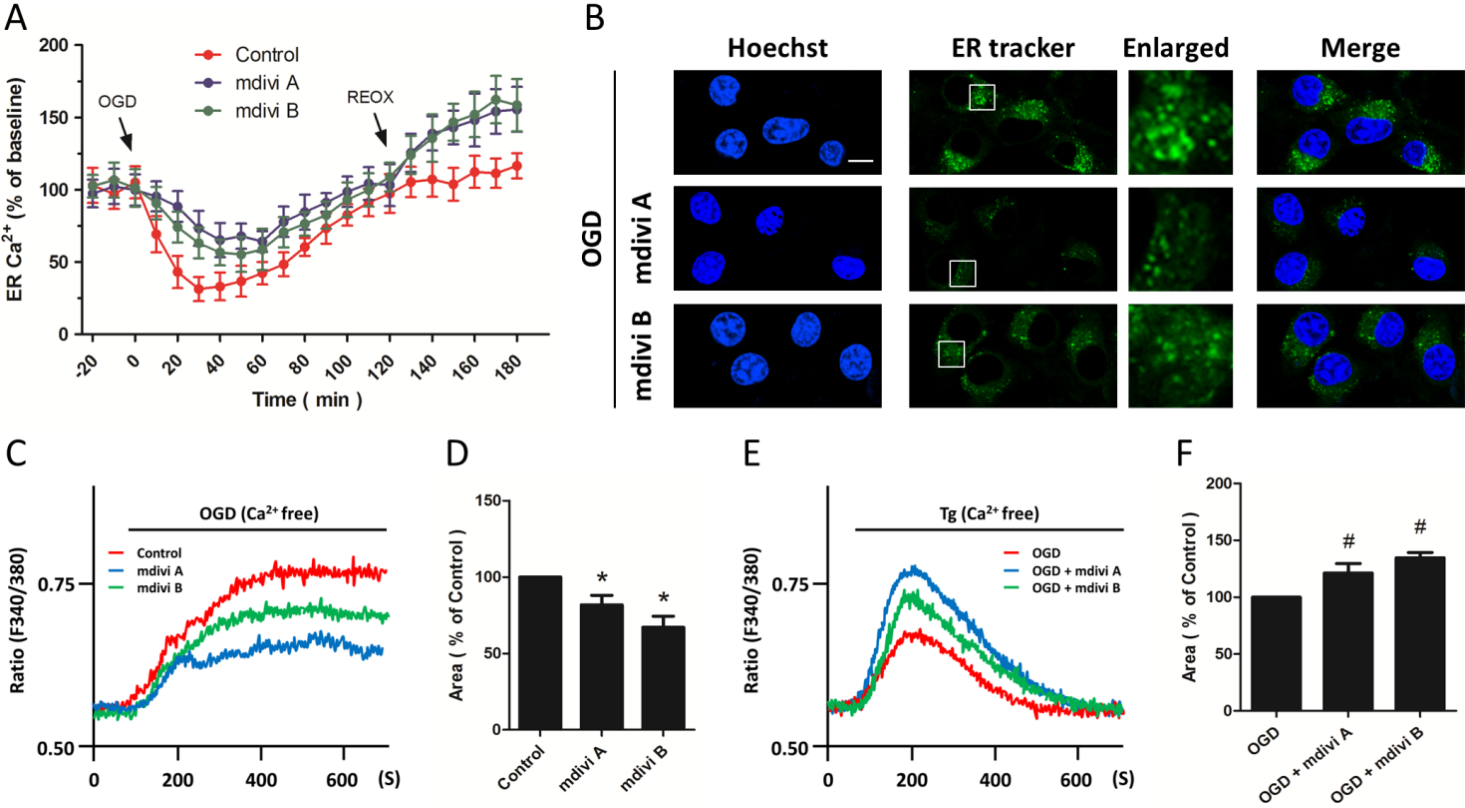
Effects of Drp-1 inhibitors on ER Ca^2+^ release. PC12 cells were pretreated with mdivi A (50 μM) or mdivi B (50 μM) for 30 min before OGD injury. The ER Ca^2+^ concentration was measured up to 180 min after OGD initiation (**A**); the morphological changes of ER were determined by ER tracker staining (**B**), scale bar: 10 μm; After pretreatment with mdivi A or mdivi B, PC12 cells were injured by OGD in Ca^2+^-free solution. ER Ca^2+^ release was measured (**C**) and calculated (**D**); After pretreatment with mdivi A or mdivi B and OGD insult, PC12 cells were treated with 200 nM thapsigargin (Tg) in Ca^2+^-free solution to induce ER Ca^2+^ release. Changes of Ca^2+^ concentration were measured (**E**) and calculated (**F**). The data were represented as means ± SD from five experiments. Each experiment in (**C**) and (**E**) is the average of at least 50 cells. * *p* < 0.05 *vs.* control. ^#^ *p* < 0.05 *vs.* OGD.

We further investigated whether the ER Ca^2+^was affected (Figure 5A). OGD insults resulted in a rapid decrease in ER Ca^2+^ that slowly returned to the baseline within 120 min. Pretreatment with mdivi A or midvi B significantly inhibited ER Ca^2+^ release and improved ER Ca^2+^ recovery during OGD, whereas the ER Ca^2+^ was higher in Drp-1 inhibitors pretreated cells after reoxygenation compared to that in control cells, indicating an inhibited ER Ca^2+^ release during OGD and an enhanced ER Ca^2+^ buffering capacity during reoxygenation after Drp-1 inhibition in OGD injured cells. We also detected the morphological changes of the ER using ER tracker staining (Figure 5B). The results showed that OGD-induced increase in ER dilation and some vacuoles formed from the destruction of ER structural integrity were prevented by Drp-1 inhibitors, suggesting the preservation of ER morphology induced by Drp-1 inhibitors. We also monitored Ca^2+^ changes in Ca^2+^-free solutions to reflect the ER Ca^2+^ release after OGD initiation (Figure 5C). As shown in Figure 5D, the OGD-induced ER Ca^2+^ release, as evidenced by Ca^2+^ rise induced by OGD in Ca^2+^-free solutions, was obviously inhibited by mdivi A or mdivi B pretreatment. Figure 5E shows representative Ca^2+^ rise traces induced by thapsigargin (Tg) in the absence of Ca^2+^ into the extra-cellular solution after inhibitors pretreatment and OGD, which indicated the residuary Ca^2+^ in ER after OGD-induced ER Ca^2+^ release. The residuary Ca^2+^ in mdivi A or mdivi B pretreated OGD injured PC12 cells was higher than that in OGD-injured cells (Figure 5F), indicating that Drp-1 inhibitors significantly preserved the ER Ca^2+^ after ischemic neuronal injury.

### 2.6. Effects of Drp-1 Inhibitors on ER Ca^2+^ Release

We further investigated whether the ER Ca^2+^ was affected (Figure 5A). OGD insults resulted in a rapid decrease in ER Ca^2+^ that slowly returned to the baseline within 120 min. Pre-treatment with mdivi A or midvi B significantly inhibited ER Ca^2+^ release and improved ER Ca^2+^ recovery during OGD, whereas the ER Ca^2+^ was higher in Drp-1 inhibitors pretreated cells after re-oxygenation compared to that in control cells, indicating an inhibited ER Ca^2+^ release during OGD and an enhanced ER Ca^2+^ buffering capacity during reoxygenation after Drp-1 inhibition in OGD injured cells. We also detected the morphological changes of the ER using ER tracker staining (Figure 5B). The results showed that OGD-induced increase in ER dilation and some vacuoles formed from the destruction of ER structural integrity were prevented by Drp-1 inhibitors, suggesting the preservation of ER morphology induced by Drp-1 inhibitors. We also monitored Ca^2+^ changes in Ca^2+^-free solutions to reflect the ER Ca^2+^ release after OGD initiation (Figure 5C). As shown in Figure 5D, the OGD-induced ER Ca^2+^ release, as evidenced by Ca^2+^ rise induced by OGD in Ca^2+^-free solutions, was obviously inhibited by mdivi A or mdivi B pretreatment. Figure 5E shows representative Ca^2+^ rise traces induced by Tg in the absence of Ca^2+^ into the extracellular solution after inhibitors pretreatment and OGD, which indicated the residuary Ca^2+^ in ER after OGD-induced ER Ca^2+^ release. The residuary Ca^2+^ in mdivi A or mdivi B pretreated OGD injured PC12 cells was higher than that in OGD-injured cells (Figure 5F), indicating that Drp-1 inhibitors significantly preserved the ER Ca^2+^ after ischemic neuronal injury.

## 3. Discussion

Mitochondrial morphology and function are governed by the delicate balance between frequent fusion and fission events, as well as by interactions with the cytoskeleton [8]. Drp-1 (Dnm-1 in yeast) is a conserved dynamin GTPase super-family protein that forms higher order structures upon binding to membranes. Drp-1 exists primarily as dimers/tetramers in the cytosol, and it assembles into larger oligomeric structures at the fission sites, wraps around the mitochondria, and then severs the mitochondrial membrane by GTP hydrolysis during mitochondrial fission process [9]. Inhibition of mitochondrial fragmentation through activation of fusion-associated proteins, such as Mfn-1/2 and Opa-1, has been demonstrated to exert protective effects against apoptosis progression [8,10]. However, the role of Drp-1-mediated mitochondrial fission event in apoptotic cell death remains controversial. Previous studies showed that inhibition of Drp-1-dependent mitochondrial fission by specific targeted siRNA reduced cancer cell proliferation and increased apoptosis in both human lung and colon cancer cells [11,12]. Recent experiments using neuron-specific *Drp-1* knockout mice or primary cultured neuronal cell from *Drp-1* knockout embryos have revealed that Drp-1 deficiency resulted in unusually shaped mitochondria with compromised intracellular movement, which leads to neuronal apoptosis [7,13]. In contrast to the Drp-1 function in apoptosis described above, numerous studies demonstrated that Drp-1 interact with Bcl-2 family proteins to mediate mitochondrial dysfunction under neuro-pathological conditions. For example, cytosolic Bax targeted the mitochondrial outer membrane and co-localized with Drp-1 and Mfn-2 at mitochondrial sites where fission subsequently occurred [14]. Drp-1 was shown to interact with Bax to form complexes at mitochondrial fission sites, mediating the outer mitochondrial membrane permeabilization and cytochrome c release [15]. In addition, modifications altering Drp-1 activity and followed mitochondrial fragmentation have been linked to human neuro-degenerative pathologies, including Alzheimer’s, Parkinson’s and Huntington’s disease [10,16]. In the present study, we found that mdivi A and mdivi B reduced the number of TUNEL-positive cells, inhibited cytochrome c release, and prevented the loss of MMP after OGD insult, indicating the anti-apoptotic activity of Drp-1 inhibition in our *in vitro* ischemia models. It is well characterized that mitochondrial fission is regulated by post-translational modifications of Drp-1, such as phosphorylation, *S*-nitrosylation, ubiquitination and *O*-GlcNAcylation, in response to diverse cellular stimuli [17]. Experiments to address whether these multi-site post-translational modifications on Drp-1 are involved in mitochondrial dysfunction in ischemic neuronal injury are currently under progress.

One of the key events that cause mitochondrial injury is an abnormal increase in intracellular Ca^2+^ [18]. Transient cerebral ischemia is accompanied by a gradual rise in intracellular Ca^2+^, by calcium sequestration in mitochondria, and followed by mitochondrial bioengergetic dysfunction. The direct and indirect effects of Ca^2+^ on mitochondrial structure and function, such as activation of degradative enzymes, uncoupling of oxidative phosphorylation, and release of matrix metabolites, can lead to either necrotic or apoptotic cell death [19]. Neuronal death by glutamate-induced excite-toxicity is inhibited when cerebellar granule neurons are treated with mitochondrial poisons that block energy-dependent mitochondrial Ca^2+^ uptake [20]. In the present study, OGD insult was found to initiate intracellular Ca^2+^ elevation coupled with mitochondrial Ca^2+^ uptake in PC12 cells. Thus, it could be speculated that OGD insult leads to intracellular calcium overload, and in turn increases mitochondrial Ca^2+^ uptake, possibly through structural changes in mitochondrial permeability transition proteins [21]. However, it is noteworthy that mdivi A and mdivi B significantly attenuated mitochondrial Ca^2+^ uptake, but had no effect on intracellular Ca^2+^ overload, indicating a cytoplasmic Ca^2+^-independent mechanism of mitochondrial Ca^2+^ uptake in ischemic neuronal injury.

The most well-characterized organelle contact sites are those between the ER and mitochondria, which have been shown to be involved in the regulation of lipid synthesis, mitochondrial biogenesis and intracellular Ca^2+^ homeostasis [22]. Ca^2+^ can be released from the ER to mitochondria at contact sites by stimuli, and this seems to play an important role in regulating mitochondrial function, division and apoptotic cell death. Several pro-apoptotic stimuli, such as H_2_O_2_, C_2_-ceramide and arachidonic acid, induced movement of Ca^2+^ from ER to mitochondria, leading to Ca^2+^ overload and caspase-mediated apoptosis [23]. Bax and Bak, two pro-apoptotic members of the Bcl-2 family facilitated mitochondrial outer-membrane permeabilization (MOMP) when Ca^2+^ is released from ER to mitochondria during apoptosis [24]. Drp-1 has been demonstrated to play an important role in MOMP induced cytochrome c release during apoptosis [25]. Conversely, the anti-apoptotic protein Bcl-2 was shown to reduce mitochondrial Ca^2+^ uptake by decreasing the releasable ER Ca^2+^ pool [26]. In the present study, we found that Drp-1 inhibitors mdivi A and mdivi B inhibited ER Ca^2+^ release during OGD and preserved ER Ca^2+^ buffering capacity during reoxygenation. These data were further confirmed by immune blot analysis showing that Drp-1 inhibitors prevented the ER dilation and vacuoles formation induced by OGD, and by calcium imaging in Ca^2+^-free solutions showing that mdivi A and mdivi B decreased the releasable ER Ca^2+^ pool after ischemic toxicity.

## 4. Experimental Section

### 4.1. Cell Culture

Rat PC12 cells (adrenal gland; pheochromocytoma) were obtained from the Shanghai Institute of Biochemistry and Cell Biology (SIBCB, Shanghai, China), Chinese Academy of Sciences (CAS, Shanghai, China). The cells were grown in Dulbecco’s Modified Eagle’s medium (DMEM, Gibco, Gaithersburg, MD, USA) plus 10% fetal bovine serum and 1% antibiotics (penicillin/streptomycin, Gibco, Gaithersburg, MD, USA) in a humidified incubator with 5% CO_2_ and 95% air. Induction of OGD was performed 24 h after seeding of the cells.

### 4.2. OGD

To initiate OGD, culture medium was removed and rinsed with phosphate buffered saline (PBS, Gibco, Gaithersburg, MD, USA) for three times. The cultured PC12 cells were placed into a specialized, humidified chamber containing 5% CO_2_, 95% N_2_ at 37 °C with glucose-free DMEM. After 2 h challenge, cells were removed from the anaerobic chamber, and the culture medium was replaced with normal culture medium with glucose. The cells were maintained for further 24 h at 37 °C in a humidified 5% CO_2_ incubator to generate reperfusion/re-oxygenation insult.

### 4.3. Cell Viability Assay

The cell viability assay was performed with Cell Proliferation Reagent WST-1 according to the manufacturer’s instructions (Roche, Shanghai, China). Cells were cultured in micro-plates in a final volume of 100 μL culture medium per well. After treatment, 10 μL of Cell Proliferation Reagent WST-1 was added to each well and incubated for 4 h at 37 °C and 5% CO_2_. After thorough shaking for 1 min on a shaker, the absorbance of the samples was measured against a background control as a blank with the enzyme-linked immunosorbent assay (ELISA) reader. Results are presented as a percentage of the control.

### 4.4. LDH Release Assay

LDH release into the culture medium was detected with a diagnostic kit (Jiancheng Bioengineering, Nanjing, China) according to the manufacturer’s instructions. Briefly, 50 μL of supernatant from each well was collected to assay LDH release. The samples were incubated with NADH and pyruvate for 15 min at 37 °C, and the reaction was stopped by adding 0.4 M NaOH. The activity of LDH was calculated from the absorbance at 440 nm, and background absorbance from culture medium that was not used for any cell cultures was subtracted from all absorbance measurements. The results were normalized to the maximal LDH release, which was determined by treating control wells for 60 min with 1% Triton X-100 (Sigma, St. Louis, MO, USA) to lyse all cells.

### 4.5. TUNEL Staining

Briefly, PC12 cells were seeded on 1.5 cm glass slides at a density of 3 × 10^5^ cells/cm^2^. Twenty-four hours after OGD initiation, cells were fixed by immersing slides in 4% methanol-free formaldehyde solution for 20 min at room temperature and permeabilized with 0.2% Triton X-100 for 5 min. Cells were labeled with fluoresce in TUNEL reagent mixture for 60 min at 37 °C according to the manufacturer’s suggested protocol. After that, slides were examined by fluorescence microscopy (Nikon, Tokyo, Japan) and the number of TUNEL-positive (apoptotic) cells was counted.

### 4.6. Measurement of Intra-Cellular ROS

Briefly, PC12 cells were incubated with DCF-DA (10 μM) for 1 h at 37 °C in the dark, and then re-suspended in PBS. Intracellular ROS production was detected using the fluorescence intensity of the oxidant-sensitive probe 2,7-dichlorodihydrofluoresce (Pierce, Rockford, IL, USA) in diacetate (H2DCFDA, Pierce, Rockford, IL, USA) in an Olympus BX60 microscope (Olympus, Tokyo, Japan) and fluorescence was read using an excitation wavelength of 480 nm and an emission wavelength of 530 nm.

### 4.7. Quantification of Cytochrome c Release

Cytochrome c release into the cytoplasm was assessed after sub-cellular fraction preparation. PC12 cells were washed with ice-cold PBS for three times and lysed with a lysis buffer (Gibco, Gaithersburg, MD, USA) containing protease inhibitors. The cell lysate was centrifuged for 10 min at 750× *g* at 4 °C, and the pellets containing the nuclei and unbroken cells were discarded. The supernatant was then centrifuged at 15,000× *g* for 15 min. The resulting supernatant was removed and used as the cytosolic fraction. The pellet fraction containing mitochondria was further incubated with PBS containing 0.5% Trition X-100 for 10 min at 4 °C. After centrifugation at 16,000× *g* for 10 min, the supernatant was collected as mitochondrial fraction. The levels of cytochrome c in cytosolic and mitochondrial fractions were measured using the Quantikine M Cytochrome C Immuno assay kit obtained from R&D Systems (Minneapolis, MN, USA).

### 4.8. Measurement of Mitochondrial Membrane Potential (MMP)

MMP was measured using the fluorescent dye rhodamine 123 (Rh123, Pierce, Rockford, IL, USA) as reported previously [27]. Rh123 was added to PC12 cells to achieve a final concentration of 10 μM for 30 min at 37 °C after the cells had been treated and washed with PBS. Hoechst 33342 (10 μg/mL, Sigma, St. Louis, MO, USA) was used to stain nucleus. The fluorescence was observed using an Olympus BX60 microscope with the appropriate fluorescence filters.

### 4.9. Measurement of Mitochondrial Calcium

Cell permeable Rhod-2 AM was used as a mitochondria selective Ca^2+^ indicator. The cells were loaded with 2 mM Rhod-2 AM for 30 min before various treatments, and then washed three times. The fluorescence intensities were immediately analyzed with a Nikon inverted fluorescence microscope. Results are presented as a fold of the control.

### 4.10. Calcium Imaging

Intracellular and ER calcium concentrations were measured by use of the ratio metric Ca^2+^ indicator Fura-2-AM (Sigma, St. Louis, MO, USA). PC12 cells grown on glass slides were loaded with 5 μM Fura-2-AM for 45 min before OGD at room temperature. Cells were then placed in an open-bath imaging chamber containing Dulbecco’s NaCl/P_i_ solution supplemented with 20 mM glucose. With a Nikon inverted fluorescence microscope, cells were excited at 345 and 385 nm, and the emission fluorescence at 510 nm was recorded. To determine ER Ca^2+^, the plasma membrane was permeablized with 30 s exposure to saponin (3.0 μg/mL) to eliminate the cytosolic fura-2 signal. This treatment caused a decrease in cytosolic mag-fura-2 fluorescence but an increase in the ratio of 345 nm/385 nm, which reflects fura-2 in ER. Images were collected and analyzed with METAFLUOR image-processing software (Scion Corporation, St. Louis, MO, USA). The Ca^2+^ concentration values were then calculated, and Ca^2+^-insensitive fluorescence was subtracted from each wavelength before calculations were performed.

### 4.11. ER Tracker Staining

To visualize the morphological changes of ER, ER tracker (Invitrogen, Carlsbad, CA, USA) was added to samples for 30 min prior to fixation with 4% paraformaldehyde (Sigma, St. Louis, MO, USA). Hoechst 33342 (10 μg/mL) was used to stain nucleus. Slides were observed by using an Olympus BX60 microscope with the appropriate fluorescence filters.

### 4.12. Measurement of ER Calcium Release

Cultured PC12 cells grown on glass slides were loaded with 5 μM Fura-2 AM in control solution for 45 min before OGD at room temperature. The cover slips were then gently washed to remove the extracellular dye and were placed in a cuvette filled with pre-warmed Ca^2+^-free solution. OGD insult and 200 nM thapsigargin (Tg) was used to induce ER Ca^2+^ release. Excitation wavelength was alternated between 340 and 380 nm and the emitted fluorescence was collected at 510 nm. Results are presented as the ratio of 340 nm/380 nm fluorescence.

### 4.13. Statistical Analysis

Statistical analysis was performed using SPSS 16.0 (Scion Corporation, St. Louis, MO, USA), a statistical software package. Statistical evaluation of the data was performed by one-way analysis of variance (ANOVA) followed by Bonferroni’s multiple comparisons. A value of *p* < 0.05 was considered statistically significant.

## 5. Conclusions

In conclusion, our results demonstrate that Drp-1 inhibitors significantly ameliorate OGD-induced neuronal apoptosis and mitochondrial dysfunction. This is achieved, at least in part, by reducing mitochondrial Ca^2+^ uptake through regulating ER Ca^2+^ release. Our report therefore provides new insights into the therapeutic action and the underlying mechanism of Drp-1 inhibitors to ameliorate ischemic neuronal injury. As potential neuroprotective agents, the beneficial effects of Drp-1 inhibitors when used to treat ischemic stroke warrant further animal researches and clinical investigations.
